# Alexithymia, intolerance to uncertainty and mental health difficulties in adolescents with Type 1 diabetes mellitus

**DOI:** 10.1186/s13052-024-01647-4

**Published:** 2024-05-17

**Authors:** Emanuele Maria Merlo, Rita Tutino, Liam Alexander MacKenzie Myles, Maria Carmela Lia, Domenico Minasi

**Affiliations:** 1 Department of Biomedical and Dental Sciences and Morphofunctional Imaging, University of Messina, Messina, Italy; 2 Pediatric Unit of Ospedali Riuniti Presidium, Grande Ospedale Metropolitano Bianchi Melacrino Morelli, Reggio Calabria, Italy; 3 Department of Experimental Psychology, University of Oxford, Oxford, Oxfordshire UK

**Keywords:** Alexithymia, Clinical psychology, Chronic conditions, Diabetes, Mental health, Type 1 diabetes mellitus, T1DM, Uncertainty

## Abstract

**Background:**

Type 1 Diabetes Mellitus (T1DM) represents a serious chronic condition affecting a wide number of people. Discussion of the physical issues associated with T1DM pervades the literature, however, there is less discussion of the psychological consequences. Mental health difficulties, alexithymia and uncertainty are present in this population, and known to be harmful for the onset, maintenance and worsening of T1DM. This study aimed to evaluate the presence of these phenomena in people with T1DM.

**Methods:**

105 participants aged between 11 and 17 years old (M: 13.88; SD: 2.16) affected by T1DM were included in the sample. To assess the presence of mental health difficulties, SAFA scales (Depression, Anxiety and Somatic symptoms) were included in the protocol together with TAS-20 and IUS-12, which evaluate the presence and role of alexithymia and intolerance to uncertainty in the sample, respectively.

**Results:**

A concerning presence of anxiety, depression and somatic symptoms was found in the sample. Mental health difficulties appeared to be consistently present in the sample, often overcoming pathological thesholds. Alexithymia and uncertainty were also common, highlighting their role in T1DM.

**Conclusions:**

Active mental health difficulties together with high rates of alexithymia and intolerance to uncertainty were prevalent in the sample of adolescents with diabetes.

**Supplementary Information:**

The online version contains supplementary material available at 10.1186/s13052-024-01647-4.

## Introduction

The literature suggests that various psychological and mental health variables impact complex medical conditions [1–6]. Among the relevant studies in this field, some authors focus in particular on the role of psychological variables in the field of medicine. In particular, Fava and colleagues [1] suggest how the manuals and guidelines that refer to the conditions that have emerged as influenced by psychological functioning should include dedicated spaces. Specifically, in line with the studies of Levenson and Linton [2, 3], there is a need for assessment and treatment of psychological factors that interfere with clinical practice, worsening the patients’ quality of life and the conditions from which they suffer.

Some studies focus in particular on specific pathological domains, such as the cardiovascular one [4], others consider a broader field in which various systems are interfered by mental health difficulties and factors known as psychosomatic [5]. With reference to this last point, it seems clear that studies in the literature highlight the multiplicity of systems affected and the consequences due to these phenomena. The extension of this knowledge concerns an ever-increasing number of pathologies, including type 1 and 2 diabetes mellitus (T1DM and T2DM), whose understanding in terms of psychological manifestations and role is particularly relevant [7–19].

In particular, recent studies have highlighted important factors related to children and adolescents suffering from Type 1 Diabetes, highlighting important associations between glycaemic control, sociodemographic status and psychological conditions, as well as in relation to relations with primary Figs. (7,8). In particular, Andrade and colleagues [7] highlight a negative association between psychological suffering and the management of diabetes (studied through glycated haemoglobin as a fundamental reference variable). Similarly, Barone and colleagues [8] highlight the need to intervene with respect to primary relationships and attachment figures, particularly in adolescence.

It is understandable how these phenomena can interfere with glycaemic control, as the need for control in T1DM over glycaemia is fundamental. Some studies have focused on sociodemographic [7] and relational variables [8], highlighting that low years of education and so-called ‘dysfunctional’ primary relationships decrease the chances of compliance with T1DM treatment. Adolescence, in particular, is characterized by significant variations in terms of development [9–11], which may impact the management of diabetes; thus, a distinction between the paediatric and adult population is necessary. A particularly interesting study by van Duinkerken and colleagues [20] illustrates that the impact of socio-demographic differences, such as working life, parenting, couple size and mature relationships, for adults, while in children the psychological difficulties experienced are more influenced by affective difficulties such as anxiety, depression and cognitive difficulties. It is important to consider, as suggested by van Duinkerken, that the number of studies conducted in adults is consistently lower than those dedicated to developing-age participants, given the age at which diagnosis is usually set for type 1 diabetes. It is known that variables such as development and psychological maturation processes can interfere on the awareness of illness, on the cognition of the necessary diagnostic and pharmacological procedures, as well as on adherence to treatments [12–15]. Fisher and colleagues [9] have long identified that there are significant psychological difficulties for people suffering from diabetes mellitus. The authors compared populations of adults and participants of developing age, specifying that it was particularly important to focus attention on stressors, particularly accidents, on the management of pathology. Furthermore, Greydanus et al. [10] specified how it was particularly important to be able to realize which variables influenced the management of the pathology in order to avoid further outcomes and chronic diseases. These studies, dating back several years ago, already highlighted how psychological factors influenced patients’ experiences, to the point that further studies clarified how emotional stress constituted particular needs for these individuals [11].

In particular, psychological factors, which have also been suspected for a long time to impact physical health conditions, emerge as particularly important in glycaemic control management and treatment compliance/adherence [21–28]. Some authors clearly specify psychological difficulties related to diabetes and how they constitute opportunities to reduce adherence to treatments, highlighting the factors primarily involved in the phenomenon and often unconscious matrix and therefore not accessible to the consciousness of the individuals [12, 13, 21]. Particularly interesting are those studies that focus instead on the role of psychological factors compared to onset diabetes [14, 16, 18, 19]. Several contributions specify etiological dynamics that would stimulate the pathophysiology of diabetes. These studies identify in the affective, socio-relational and often unconscious dynamics underlying to stimulate the pathophysiology of the condition. Among the various phenomena involved in the management of therapies, illness denial, uncertainty, alexithymia and affective mental health difficulties insistent on the ideation of patients, appear more relevant than ever in clinical settings [29–50].

Even if the growing number of studies indicate with increasing precision what the target phenomena are, there is an evident need to establish a comparison between studies and secondly to implement existing knowledge through new and more reliable clinical tools [20, 51–58]. Based on the known literature, the application of the uncertainty intolerance (UI) model to type 1 diabetes, together with knowledge on alexithymia and the affective dynamics of the participants, would constitute an example of innovative research useful for the evaluation of these not fully conscious phenomena [59–64]. Alexithymia has been shown to be present in many pathological conditions and represented in the general population [59, 65–67]. In particular, it is known that this phenomenon is common in the general population, representing a variable diffused at different levels (dimensional approach). With particular reference to T1DM, it is known how this phenomenon can interfere with physical condition [68]. In particular, alexithymia has emerged in many important studies as present in participants affected by T1DM, involved in fundamental areas such as glycaemic control [69, 70], adherence to treatments and the aforementioned psychological and mental health difficulties dynamics [71–73]. Similarly, intolerance to uncertainty can be considered as a common phenomenon for populations, but it can pose a threat to the health of participants when levels exceed an acceptable threshold. In particular, some studies related to T1DM have highlighted the role of intolerance to uncertainty. In a 2019 study, Perez and colleagues [74] highlight how intolerance to uncertainty can be a limit in the individual’s adaptation to existential conditions. The method used by the authors included 29 parents of participants suffering from Type 1 Diabetes, so the related experience was mediated by representations of parents. A recent review by Gibson and colleagues [75] suggests it is necessary to study in depth the role of the variable Uncertainty in participants affected by this condition. In addition to suggesting the absence of data consistent with the literature, the researchers also point out that the available data refer more to physical health outcomes such as hba1c in people with type 2 diabetes. Recently, thanks to an exploratory and differential study [64] it has been possible to highlight how people affected by Type 1 Diabetes in adolescence have high levels of Uncertainty, Alexithymia and mental health difficulties in accordance with the studies mentioned above. In particular, positive correlations emerged between alexithymia (well-studied in the literature for T1DM) and uncertainty. The correlations were positive to indicate the same direction taken by the phenomena. In addition, variables such as age and years of education turned out to take significant and negative directions with respect to alexithymia, demonstrating how the mentalization acquired with growth and years of education can represent important variables with respect to alexithymia. However, there were no significant relationships between age and years of education predictors and uncertainty variables. Other studies have shown that uncertainty often addresses the issue of chronicity, given the duration and temporality of the chronic condition [76] and also in this case we believe that the issue should be considered with reference to the degree of development of the participants. It is clear that the impact of the diagnosis and the prolonged psychological distress represented by the chronicity of the disease are for the subject occasion of the onset of other conditions. In the strict sense, Carpentier and colleagues [77] highlight how the issue can be a critical existential point in the life of participant to caregivers, such as to have to be treated and treated in order to limit the impact. In both cases alexithymia and intolerance to uncertainty represent common phenomena to the population, whose presence does not indicate pathology per se. In this sense, the assessment of these phenomena must be considered in dimensional terms and declined to any particular diagnoses that could constitute an impact. Recent research also emphasizes the impact of these diagnoses on family members and relatives, which is expressed differently relative to specific age groups [78], in childhood [79, 80], adolescence [81, 82] and adulthood [83–88]. Gender is an important variable in terms of differential and presentation of the above variables compared to Type 1 Diabetes. In particular, some studies have referred to gender to clarify the role and dynamics of gender. Enzlin and colleagues [89] highlighted how women reported more depressive symptomatology than men and how significant gender differences were also found in psychological adjustment to diabetes. The authors make it clear that this variable is often neglected in literature and that there is a need to produce studies that clarify its role and scope. In most of the cases, in fact, it is suggested a greater propension for the female population in the onset of affective symptoms [90], variables considered essential in the management of diabetes. Other studies [91] suggest high overall levels of distress in people suffering from Type 1 Diabetes Mellitus, with particular reference to the female population. In detail, there was higher physician-related distress and lower regimen-related distress than males. The authors referred to the problem of a greater propensity of the female population to report the affective symptomatology [92], compared to the male population [93]. The fact that these data refer almost exclusively to adult populations reinforces the need to produce further data in order to understand the dynamics involved in adolescent and infant populations.

In this sense, the need to deepen the understanding in this area is evident. The suggested in-depth analysis would constitute an opportunity for the scientific structuring of targeted interventions supported by a comprehensive understanding of intrapsychic processes typical in this population.

### Study hypotheses

The purpose of this study was to highlight the facets associated with various factors in T1DM, including socio-demographic characteristics, affectivity, presence of mental health difficulties, emotional functioning, difficulty in recognizing emotional experience and uncertainty related to type 1 diabetes mellitus.

In order to elucidate T1DM-specific symptomatology, we developed the below hypotheses concerning the presence of difficulties related to affective dynamics, uncertainty, related correlations and differences in T1DM.

#### Hypothesis 1:

Presence of mental health difficulties components, alexithymia and intolerance to uncertainty;

#### Hypothesis 2:

Significant correlation among sociodemographic variables (age, years of education, age of diagnosis and diabetes duration) and variables related to anxiety, depression and somatic symptoms;

#### Hypothesis 3:

Significant correlation between variables related to alexithymia and intolerance to uncertainty;

#### Hypothesis 4:

Statistically significant differences among age and gender groups considering alexithymia and intolerance to uncertainty.

## Methods

### Procedure and participants

The sample consisted of 105 participants, 63 of whom were female (60%), aged between 11 and 17 years old (M: 13.88; SD: 2.16; Years of education, M: 8.06, SD: 3.02) affected by Type 1 Diabetes Mellitus (Age of diagnosis, M:7.79, SD: 3.35; Diabetes duration, M:6.08, SD: 3.75).

The research was carried out at the Pediatric Unit of the Ospedali Riuniti of Reggio Calabria, Italy, and at University of Messina, Messina, Italy. Patients were recruited during normal clinical activities of the Pediatric Unit directed by DM. DM asked participants and their legal guardians about their willingness to participate in the cross-sectiona study. The participants involved in the study were all patients of the UOC Paediatrics’ Clinic mentioned above. The inclusion criteria concerned the diagnosis of T1DM in childhood, the absence of comorbidity or other medical conditions or previous childhood psychiatric diagnosis. All participants included in the study and their guardians agreed to participate. The success rate for enrolling participants was 95%, while 5% of participants refused and were not interested in the study. Written informed consent was obtained from all participants or if participants were under 16 years old, from a parent and/or legal guardian. Participants and parent/legal guardians were informed about the anonymous nature of data collection, consistent with the 1964 Declaration of Helsinki. The study was approved by local Ethical Committee (Comitato Etico Regionale– Sezione Area Sud, Grande Ospedale Metropolitano “Bianchi-Melacrino-Morelli” of Reggio Calabria, N°: 19-2022, from 27/4/2022 onwards). All methods were performed in accordance with the ethical standards as laid down in the Declaration of Helsinki and its later amendments or comparable ethical standards. Assuming an incidence of mental disorders in the adolescent population of 16% [94], an incidence in the sample under examination (adolescents suffering from T1DM) of 30% [95], considering an alpha significance level of 5%, the minimum number of participants to be enrolled in order to have a statistical power of 85% is equal to 73 participants. Therefore, a total sample of 105 participants were enrolled. The administration of the protocols took place from 3 May 2022 to 30 April 2023. Protocol administration was paper and pencil.

### Instruments

#### Socio-demographic characteristics

The socio-demographic characteristics of the sample were obtained through a self-administration questionnaire examining age, gender, years of education and age at diagnosis of type 1 diabetes mellitus.

#### Intolerance to uncertainty scale − 12

The Intolerance of Uncertainty Scale 12 (IUS-12) is a scale measuring intolerance to uncertainty. IUS-12 is a 2-factor scale that constitutes the abridged version [96] of a previous 27-item scale (IUS-27) [97, 98]. The two factors were described as Prospective and Inhibitory dimensions of IU [96, 99]. The IUS-12 is a self-report tool based on 12 items with a 5-point Likert scale [100]. According to Bottesi and colleagues [101], “uncertainty intolerance can be thought of as the individual’s dispositional inability to tolerate the aversive response triggered by the perceived absence of salient, key, or sufficient information, and is sustained by the associated perception of uncertainty”. The original version [102], which was reduced by Carleton et al. [96], provided 12 weighted items belonging to two factors: prospective anxiety and inhibitory anxiety. With reference to this two-factor structure, strong psychometric properties of the SUI have been reported, in addition to a high correlation with original SUI (*r* =.96). Total and subscale scores demonstrated consistent construct validity, internal reliability, and test-retest reliability (Cronbach’s α of 0.91, total scale, 0.85 for both subscale scores, *r* =.77) [96, 103]. The extent of studies on the SUI model in adolescents is limited compared to the application in adults. Previous studies have demonstrated the applicability of the model [104]. In our study the Cronbach’s alphas were 0.742 for IUS-12 Total score, 0.627 for IUS-12 Prospective anxiety and 0.659 for IUS-12 Inhibitory anxiety.

#### Toronto alexithymia scale– 20

The Toronto Alexithymia Scale (TAS-20) [51] is a well-known 20-item self-assessment tool, based on a 5-point Likert scale. The original version of the TAS-20 demonstrated an internal consistency of 0.81 (Cronbach’s α), reporting a three-factor structure accounting for 31% of the total variance: Difficulty identifying feelings (0.78), Difficulty describing feelings (0.75) and Externally oriented thinking (0.66). In 1996, Bressi and colleagues [105] published a cross-validation of the TAS-20, performing the psychometric analyses for both clinical and non-clinical participants. In detail, the scores of the α coefficient obtained with the non-clinical sample were 0.75 for the total scale, 0.77, 0.67 and 0.52 respectively for the first, second and third factors; the scores of the clinical sample were 0.82 for the full scale, 0.79, 0.68 and 0.54 for the three factors. Further studies [106, 107] have analyzed the psychometric properties of the scale, highlighting the good consistency and reliability of the three-factor structure. For the current study Cronbach’s alphas were 0.760 for the TAS-20 Total Score, 0.789 for TAS-20-DIF- Difficulty identifying feelings, 0.607 for TAS-20-DDF-Difficulty describing feelings and 0.665 for TAS-20-EOT-Externally oriented thinking.

#### SAFA scales

SAFA is a psychometric test developed by Cianchetti and Sannio Fascello [108, 109] and validated in 2001. It is presented as a unitary tool that allows a preliminary and exhaustive investigation of the mental health conditions of the participants through different self-assessment scales. The test is aimed at individuals between the ages of 8 and 18 and is adapted to different levels of understanding based on age and schooling. The administration includes 6 scales, each organized into several subscales. In this case, only scales concerning Anxiety, Depression and Somatic Symptoms were considered. SAFA A (anxiety) is divided into 4 subscales: generalized anxiety, social anxiety, separation anxiety and school-related anxiety. Scale D (depression) is divided into 7 related subscales: depressed mood, anhedonia/disinterest, irritable mood, sense of inadequacy, level of self-esteem, insecurity, sense of guilt and despair. The somatic symptoms also present in mood disorders are grouped in the SAFA S, divided into the 2 subscales of somatic symptoms and hypochondriasis. Items are followed by a 3-point Likert scale ranging from “Not at all “(0) to “Entirely” [2]. For the above-mentioned validation study the Cronbach’s alphas for SAFA Anxiety were.887 for non-clinical sample and.956 for the clinical sample (test-retest Pearson *r*:.913, highly significant),.909 for non-clinical sample and.943 for the clinical sample (test-retest Pearson *r*: 881, highly significant) of SAFA Depression scale,.916 for non-clinical sample and.895 for the clinical sample (test-retest Pearson *r*:.820) for SAFA Obsession Scale,.814 for the non-clinical sample (test-retest Pearson *r*:.740, highly significant) for SAFA Psychogenic eating disorders scale,.876 for non-clinical sample and.797 for the clinical sample (test-retest Pearson *r*:.567, highly significant) of Somatic symptoms and hypochondria scale. In our case, the Cronbach’s alphas were.943 for SAFA Anxiety total score,.844 for SAFA-A Generalized anxiety,.848 for SAFA-A Social anxiety,.835 for SAFA-A Separation anxiety,.864 for School anxiety,.957 for SAFA Depression total score,.893 for SAFA D Depressed mood,.908 for SAFA D Anhedonia,.730 for SADA D Irritable mood,.891 for SADA D Low self-esteem,.737 for SAFA D Insecurity,.863 for SAFA D Sense of guilt,.915 for SAFA D Desperation, and.955 for SAFA Somatic symptoms and hypochondria total score,.948 for SAFA S Somatic symptoms and.626 for SAFA S Hypochondria.

#### Statistical analysis

Numerical data were expressed as means and standard deviations, and the categorical variables as numbers and percentages. The Spearman test was used to evaluate the correlations among variables of the involved instruments (IUS-12, TAS-20, SAFA scales). A *p* value < 0.05 was considered to be significant. The non-parametric approach was used since non-normality was verified for most of the variables examined. Kruskal-Wallis test was used to assess statistically significant differences among age and gender groups with reference to clinical scales (*p* value < 0.05). After the emergence of significant differences among age and gender groups, the Mann-Whitney test was used in order to detect significant differences among each of the quartiles (*p* value < 0.008 after Bonferroni’s correction). Statistical analyses were performed using SPSS 26 for Windows. A p-value smaller than 0.05 was considered to be statistically significant.

## Results

Descriptive statistics (mean and standard deviation) are reported in Tables 1, 2, 3, 4 and 5.

### Hypothesis 1

The first hypothesis regarded the presence of phenomena related to mental health difficulties, such as anxiety, depression, somatic symptoms (and related subscales/factors), alexithymia and intolerance to uncertainty, in the included participantssuffering from T1DM. In this case IUS-12, TAS-20 and SAFA scales were included. The results are displayed in Tables 1, 2, 3, 4 and 5.

**Table 1:** Descriptive statistics for sociodemographic, Tas-20 and IUS-12 variables

Variable	Mean	SD
TAS-20 Total Score	54.67	12.35
TAS-20-DIF- Difficulty identifying feelings	17.57	6.79
TAS-20-DDF-Difficulty describing feelings	13.55	4.22
TAS-20-EOT-Externally oriented thinking	23.55	5.83
IUS-12 Total score	34.78	8.52
IUS-12 Prospective anxiety	22.17	5.33
IUS-12 Inhibitory anxiety	12.60	4.89

TAS-20 Total score appeared to be higher than 50, demonstrating a borderline score for the entire sample. Internal factors showed consequently high scores. IUS-12 scores appeared to be high in the sample, in line with alexithymia scores. Data emerged from descriptive statistics confirming the consistent presence of this in the sample.

**Table 2:** Clinical variables (SAFA scales) for 11–13 years old male participants (25 participants)

Variable	Mean	SD
SAFA-A-Anxiety Total Score	58.08	21.06
SAFA-A-Generalized Anxiety Total Score	14.56	5.67
SAFA-A-Generalized Anxiety - Tension	5.80	3.02
SAFA-A Generalized Anxiety - Worry for the future	8.76	3.20
SAFA-A - Social Anxiety	12.64	6.08
SAFA-A - Separation anxiety	10.60	4.11
SAFA-A - School Anxiety	13.68	4.98
SAFA-D - Depression Total Score	65.44	26.26
SAFA-D Depressed Mood	8.16	4.69
SAFA-D Anhedonia	7.96	5.31
SAFA-D Irritable mood	8.32	3.26
SAFA-D Low self-esteem	8.32	5.20
SAFA-D Insecurity	7.68	3.47
SAFA-D Sense of guilt	7.64	4.32
SAFA-D Desperation	8.12	5.54
SAFA-S Somatic Symptoms Total Score	26.80	14.91
SAFA-S Hypochondria	4.84	2.74

SAFA scale’s validation study provided for mean scores obtained from pathological and non-pathological participants with reference to all foreseen mental health difficulties. These differential data allow researchers and clinicians to consider the nature of emerged scores, so that it is possible to perform a clear comparison with both groups. Anxiety scores were higher than in the non-pathological validation group. None of the variables overcame the pathological scores, but indexes were always borderline. Depression scores were always higher than in the non-pathological validation group. Anhedonia and Irritable mood scores overcome pathological group indexes. Variables referred to Somatic symptoms showed scores higher than non-pathological and pathological indexes reported in the validation studies [108].

**Table 3:** Clinical variables (SAFA scales) for 11–13 years old female participants (17 participants)

Variable	Mean	SD
SAFA-A-Anxiety Total Score	50.90	23.46
SAFA-A-Generalized Anxiety Total Score	24.00	11.54
SAFA-A-Generalized Anxiety - Tension	4.68	2.98
SAFA-A Generalized Anxiety - Worry for the future	6.86	4.23
SAFA-A - Social Anxiety	11.36	5.53
SAFA-A - Separation anxiety	10.18	4.64
SAFA-A - School Anxiety	12.40	7.66
SAFA-D - Depression Total Score	62.50	28.81
SAFA-D Depressed Mood	8.00	4.84
SAFA-D Anhedonia	9.63	5.42
SAFA-D Irritable mood	6.36	4.18
SAFA-D Low self-esteem	8.77	4.97
SAFA-D Insecurity	5.68	3.92
SAFA-D Sense of guilt	8.86	4.53
SAFA-D Desperation	9.22	5.16
SAFA-S Somatic Symptoms Total Score	30.63	14.71
SAFA-S Hypochondria	5.90	2.50

Starting from Anxiety variables (SAFA-A), all variables showed scores higher than mean scores from the validation study (non-pathological participants). With reference to Depression variables (SAFA-D), only Insecurity appeared to have a score lower than mean scores from the validation study (non-pathological participants) [108]. SAFA-D Depression Total Score, Anhedonia, Sense of Guilt and Desperation reported scores higher than validation study’s pathological group indexes. Somatic symptoms scale appeared higher than pathological participants’ group (validation studies), followed by Hypochondria reporting the same level.

**Table 4:** Clinical variables for (SAFA scales) 14–18 years old male participants (22 participants)

Variable	Mean	SD
SAFA-A-Anxiety Total Score	53.11	30.25
SAFA-A-Generalized Anxiety Total Score	13.70	6.87
SAFA-A-Generalized Anxiety - Tension	6.11	2.64
SAFA-A Generalized Anxiety - Worry for the future	7.58	4.45
SAFA-A - Social Anxiety	11.58	7.64
SAFA-A - Separation anxiety	9.41	5.75
SAFA-A - School Anxiety	12.82	8.36
SAFA-D - Depression Total Score	63.35	32.49
SAFA-D Depressed Mood	8.88	5.96
SAFA-D Anhedonia	8.05	5.56
SAFA-D Irritable mood	7.41	3.27
SAFA-D Low self-esteem	8.11	5.60
SAFA-D Insecurity	7.52	4.01
SAFA-D Sense of guilt	8.35	5.04
SAFA-D Desperation	8.35	6.06
SAFA-S Somatic Symptoms Total Score	26.64	20.21
SAFA-S Hypochondria	4.23	3.56

Anxiety scores were higher than in the non-pathological validation group. Moreover, Social Anxiety and School Anxiety were higher than in the pathological validation sample scores [108].

**Table 5:** Clinical variables for (SAFA scales) 14–18 years old female participants (41 participants)

Variable	Mean	SD
SAFA-A-Anxiety Total Score	53.17	17.24
SAFA-A-Generalized Anxiety Total Score	14.36	5.74
SAFA-A-Generalized Anxiety– Tension	6.17	2.61
SAFA-A Generalized Anxiety– Worry for the future	8.31	3.35
SAFA-A– Social Anxiety	11.75	4.99
SAFA-A - Separation anxiety	9.66	3.48
SAFA-A– School Anxiety	12.48	5.95
SAFA-D– Depression Total Score	58.21	21.35
SAFA-D Depressed Mood	7.85	4.19
SAFA-D Anhedonia	6.43	4.04
SAFA-D Irritable mood	7.78	3.53
SAFA-D Low self-esteem	7.95	3.55
SAFA-D Insecurity	7.70	3.41
SAFA-D Sense of guilt	6.39	3.82
SAFA-D Desperation	7.29	4.02
SAFA-S Somatic Symptoms Total Score	25.51	12.80
SAFA-S Hypochondria	3.48	2.84

Anxiety variables exceeded the validation study mean scores for non-pathological participants in all cases. Social anxiety scores of included participants were higher than pathological group indexes (validation studies). Considering depression, all scores showed to overcome non-pathological group indexes. In this case, none of the scores was higher than pathological indexes [108].

### Hypothesis 2

The second hypothesis concerned the presence of statistically significant correlations among sociodemographic variables and mental health variables. Considering the variable years of education, the number of years was considered at the time of the summation of the protocol. Results are presented in Table 6 and subsequently discussed.

**Table 6:** Correlation coefficients among sociodemographic and clinical variables

	Age	Years of education	Age of diagnosis	Diabetes duration
SAFA-A-Anxiety Total Score	− 0.138	− 0.202*	0.156	− 0.220*
SAFA-A-Generalized Anxiety Total Score	0.097	− 0.026	− 0.009	0.076
SAFA-A-Generalized Anxiety - Tension	0.010	− 0.083	0.023	− 0.008
SAFA-A Generalized Anxiety - Worry for the future	0.035	− 0.073	0.004	0.025
SAFA-A - Social Anxiety	− 0.116	− 0.198*	0.164	− 0.222*
SAFA-A - Separation anxiety	− 0.158	− 0.155	0.153	− 0.229*
SAFA-A - School Anxiety	− 0.150	− 0.183	0.129	− 0.217*
SAFA-D - Depression Total Score	− 0.156	− 0.192*	0.142	− 0.213*
SAFA-D Depressed Mood	− 0.065	− 0.176	0.119	− 0.122
SAFA-D Anhedonia	− 0.225*	− 0.228*	0.094	− 0.212*
SAFA-D Irritable mood	− 0.035	− 0.087	0.027	− 0.018
SAFA-D Low self-esteem	− 0.168	− 0.225*	0.128	− 0.203*
SAFA-D Insecurity	0.044	− 0.013	0.046	− 0.006
SAFA-D Sense of guilt	− 0.161	− 0.193*	0.079	− 0.151
SAFA-D Desperation	− 0.174	− 0.217*	0.146	− 0.222*
SAFA-S Somatic Symptoms Total Score	− 0.141	− 0.127	0.072	− 0.138
SAFA-S Hypochondria	− 0.267*	− 0.204*	0.156	− 0.293**

*Note* *p <.05 (two-tailed), **p <.01 (two-tailed)

With reference to correlation analyses, Table 6 reports the correlations between age, years of years of education and age of diagnosis and all included clinical variables (SAFA-A, SAFA-D, SAFA-S and related factors). All significant correlations emerged were negative. Starting with age, this appeared to be inversely correlated to anhedonia and hypochondria, suggesting a decrease of pathological affective dynamics as age increased. Considering years of education, significant inverse correlations emerged involving SAFA-A anxiety total score, social anxiety, depression total score, low self-esteem, sense of guilt, desperation and hypochondria. These significant correlations suggested the decrease of these previously mentioned pathological domains referred to years of education increase. No significant relations emerged with reference to age of the diagnosis. Finally, considering diabetes duration several significant correlations emerged. All significant correlations were negative, highlighting the decrease of mental health difficulties in the light of diabetes duration increase. The first significant and negative correlation emerged was referred to Safa a total score, followed by social anxiety, separation anxiety and school anxiety. With reference to depression scales, significant and negative correlations emerged including depression total score, anhedonia, low self-esteem and desperation. Considering somatic symptoms (SAFA-S) the only significant and negative emerged correlation was referred to hypochondria.

### Hypothesis 3

The third hypothesis concerned the presence of significant correlations among alexithymia and intolerance to uncertainty variables. Data and implications are discussed as follows.

**Table 7:** Correlation coefficients among TAS-20 and IUS-12 variables

	TAS-20Total score	TAS-20-DIF- Difficulty identifying feelings	TAS-20-DDF-Difficulty describing feelings	TAS-20-EOT-Externally oriented thinking
IUS-12 Total Score	0.361**	0.804**	0.234*	0.079
IUS-12 Prospective anxiety	0.172	0.290*	0.043	0.009
IUS-12- Inhibitory anxiety	0.402**	0.422**	0.301**	0.121

*Note* *p <.05 (two-tailed), **p <.01 (two-tailed)

Table 7 reported the correlations amongst TAS-20 and IUS-12 variables. All significant correlations were positive. Starting with TAS-20 total score, two significant indexes emerged, such that the increase of general alexithymia corresponded to an increase of uncertainty and subsequent inhibitory anxiety. Difficulty identifying feelings appeared to be consistently linked to uncertainty variables, showing positive and significant correlations. More specifically, increased difficulties in identifying feelings corresponded to increased uncertainty, prospective anxiety and inhibitory anxiety. Difficulty describing feelings showed to be in a significant positive predictor of general uncertainty and inhibitory anxiety, in line with general alexithymia. No significant correlations emerged between externally oriented thinking, a result congruent with previous literature.

### Hypothesis 4

The last hypothesis concerned statistically significant differences among age and gender groups in the light of alexithymia and intolerance to uncertainty variables. Data are represented within Tables 8 and 9, and then discussed.

**Table 8:** Kruskal-Wallis test analyses (age and gender groups compared to clinical scales)

Group		TAS-20 Total score	TAS-20-DIF	TAS-20-DDF	TAS-20-EOT	IUS-12 Total score	IUS-12 Prospective anxiety	IUS-12 Inhibitory anxiety	SAFA A	SAFA D	SAFA S
11–13 years old male	Mean	56.200	17.320	14.040	24.840	34.960	21.760	13.200	58.080	65.440	26.800
	SD	13.781	7.453	4.107	5.225	7.860	4.474	4.645	21.065	26.261	14.910
11–13 years old female	Mean	57.863	18.863	14.272	24.727	33.181	20.818	12.363	50.909	62.500	30.636
	SD	11.893	5.874	4.661	6.227	8.743	5.819	4.706	23.464	28.817	14.714
14–18 years old male participants	Mean	51.823	13.941	12.058	25.823	33.705	23.941	9.764	53.117	63.352	26.647
	SD	11.647	6.878	3.381	5.525	8.737	4.955	5.202	30.250	32.499	20.214
14–18 years old female participants	Mean	53.219	18.536	13.487	21.195	35.975	22.414	13.561	53.170	58.219	25.512
	SD	11.902	6.492	4.353	5.469	8.833	5.656	4.706	17.240	21.359	12.804
Total	Mean	54.676	17.571	13.552	23.552	34.781	22.171	12.609	53.857	61.666	27.076
	SD	12.354	6.794	4.226	5.835	8.529	5.339	4.890	21.796	25.952	14.988
*p* value		0.245	0.084	0.377	0.011*	0.501	0.291	0.036*	0.671	0.572	0.467

*p-value < 0.05

The Kruskal-Wallis test was applied with reference to age and gender as differential variables. Four groups were considered, 11–13 years old male and female participants, 14–18 years old male and female participants. The emerged significant differences emerged among the four selected groups with reference to IUS-12, TAS-20 and SAFA scales. The Kruskal-Wallis test permitted only to highlight existent statistical differences among the four groups. In order to detect the precise differences among all possible couples the Mann-Whitney test was performed and presented in the following tables. Two significant differences emerged among the four groups and were referred to TAS-20 Externally Oriented thinking (EOT) and IUS-12 Inhibitory anxiety. No significant differences appeared to emerge with reference to the SAFA scales, therefore with respect to anxiety, depression and somatic disorders.

**Table 9:** Specific differences among age and gender groups with reference to TAS-20-EOT and IUS-12 Inhibitory anxiety

	11–13 Male vs. 11–13 Female	11–13 Male vs. 14–18 Male	11–13 Male vs. 14–18 Female	11–13 Female vs. 14–18 Male	11–13 Female vs. 14–18 Female	14–18 Male vs. 14–18 Female
TAS-20-EOT	0.856	0.546	0.013	0.670	0.016	0.008*
IUS-12 Inhibitory anxiety	0.467	0.028	0.796	0.042	0.272	0.007*

*p-value < 0.008 after Bonferroni’s correction

The Mann-Whitney text allowed the differential analysis between the groups constituted by age and gender with reference to those variables where significance emerged. Specifically, the variables involved were TAS-20 Externally oriented thinking and IUS-12 Inhibitory anxiety scales. The analysis of each individual pair allowed the emergence of two significant differences, both referred to 14–18 male vs. female groups. The significant difference refers to male and female participants both in the 14–18-year-old age range. Specifically, the scores referring to male participants were higher. Considering the dynamics investigated, i.e. Externally oriented thinking, the data highlights how younger age and male sex present higher scores. The second domain studied is that of intolerance to uncertainty and in particular Inhibitory anxiety. The last significant difference concerns the two groups of participants aged between 14 and 18 years old, with higher scores in male participants. Some of the emerged values presented scores close to the corrected p-value. In these terms we can only present them as tendencies, as in the case of 11–13 male vs. 14–18 female groups considering TAS-20-EOT and 11–13 female vs. 14–18 female groups considering TAS-20-EOT.

## Discussion

The current article investigated the presence of mental health issues together with presence and role of alexithymia and uncertainty in participants suffering from T1DM. The data indicated the presence of depression, anxiety and somatic symptoms in participants with T1DM. This datum appears to be clinically concerning, considering the young age of participants involved in the study.

These phenomena appear to be in line with current studies [110, 111] as well as with recent systematic studies, meta-analyses and longitudinal studies [95, 112, 113]. In particular, anxiety and depression represent the main difficulties in adolescence [114, 115] associated with diabetes according to most recent reviews [116, 117]. Effectiveness of interventions showed its impact on these populations, encouraging their presence in the care domain [118].

In line with mental health difficulties associated with T1DM, alexithymia and uncertainty were associated with T1DM in the current sample. Starting with alexithymia, its presence was consistent in our participants (mostly borderline scores), confirming previous literature results [71, 119–121].

Mental health difficulties and psychosomatic complications related to alexithymia are well known, such that its presence must be considered a consistent risk factor for the onset and course of the pathology (T1DM). Dermatological, respiratory, cardiovascular and gastrointestinal outcomes due to the presence of alexithymia are widely discussed in literature [71, 119–124].

Results concerning the role of uncertainty in participants’ psychological functioning highlighted a clear role of alexithymia. The role of uncertainty appears to be less investigated than alexithymia with respect to physical disorders. With particular reference to diabetes, its role needs to be more extensively investigated due to a lack of knowledge in the current state of the art. Most studies reported uncertainty and alexithymia role in maladjustment, emotion processing and eating disorders [73, 125–129].

In an article of particular interest, Lumely and colleagues [73] critically analysed the role of alexithymia with respect to physical pathologies. The hypothesis accepted and carried forward by the studies also reported that alexithymia is associated with what is called a tonic physiological hyperarousal, subsequent unhealthy behaviours and a biased perception of somatic sensations including symptoms. In this sense, it is interesting to note how the positive correlation emerged between alexithymia and intolerance to uncertainty represents important data in the understanding their physical outcomes.

It is clear from this study that alexithymia is a serious influence on illness behaviour. Given the field of interest, namely Type 1 Diabetes Mellitus, the variables that affect adherence to treatments are of primary interest. Understanding the complexity of the phenomena produces a level of knowledge useful for clinical intervention. The reference to eating disorders is therefore supported by the degree of co-morbidity that includes diabetes, where it is clear that alexithymia and affective disorders produce adverse outcomes in the management of conditions. Brown and colleagues [125] report on intolerance to uncertainty within eating disorder conditions, clarifying their role in producing a consistent vulnerability for participants. The same, through their systematic review and meta-analysis, find studies that confirm the positive correlation between intolerance to uncertainty and alexithymia [124, 130]. In addition, Larkin and colleagues [126] highlight the predictive role of intolerance to uncertainty compared to somatic symptoms in healthy and pathological participants. This predictive role, related to the maladaptive outcomes of alexithymia [127] produces a strong incidence in the psychological maladjustment of participants, requiring clinical attention and evidence-based data.

In our perspective, alexithymia represents a key point in the understanding of some relevant phenomena. Difficulties in mentalizing emotions and affective dynamics may lead to the onset of physical disorders, a proposition substantiated by current and past literature. Recent contributions in the literature confirm the role of alexithymia as a predictor, in several ways. Alexithymia appeared as a strong predictor of negative outcomes in the treatment of functional and chronic pathologies, in line with what Lumely suggested [73]. The phenomenon is also treated as a predictor of poor compliance and poor outcomes related to different pathologies [131–134]. It is particularly involved in somatization processes, where the lack of processing of affective experiences is addressed to target organs. More concretely, the persistence of non-mentalized affective phenomena would produce changes in hormonal structures such as to invest target organs.

With direct reference to the neurobiological functioning linked to alexithymia, Meza-Concha and colleagues [135] recently published a clinical review purely based on the phenomenon of alexithymia. The results of the study confirmed the validity of the phenomenon also on the neurobiological level, through several studies that accurately framed phenomena of functional and structural alteration of different areas. In particular, alexithymia was previously linked to reduced interhemispheric brain connection.

In terms of what was defined as a traumatic perspective, the right prefrontal cortex and the network of predefined modes would undergo changes, first hypermetabolic (linked to dopaminergic dysregulation and glutamatergic) and then hypometabolic-dissociative (related to serotonergic and opioid dysregulation), resulting in distortion of interoceptive and emotional awareness, typical of alexithymia in its own sense.

The issue directly based on unconscious processing and traumatic experiences underlying alexithymia involves an early hypermetabolic state implying the activation of sympathetic nervous system through the start-up of neuroendocrine axis, based on corticotropin release factor, the increase in catecholaminergic and mineralocorticoid activity, to produce a dopaminergic and glutamatergic dysregulation [136]. The issue is closely linked to dissociative phenomena and experiences, as proposed by Schore [136] and colleagues, in this case it would not be dissociative phenomena in its own sense even if the pathways of neurobiological production of alterations would be similar. Other forms of study have analysed the role of mirror neurons [137, 138] in the theory of mind in the light of alexithymia, suggesting the continuity between some forms of autism and alexithymia compared to a deficient hemodynamic activity in some regions of mirror neurons system. Difficulties in recognizing and managing affective dynamics can be considered as a reflective phenomenon of alexithymia, where the impossibility to recognize emotions assume the same direction of intolerance to uncertainty. The positive correlations emerged support this common direction assumed by alexithymia and intolerance to uncertainty. In our experience the interpretation of this significant link should be linked to the shared basis by these two phenomena.

In phenomenological and dynamic terms, the absence of an object to which mental functioning is directed has been attributed to anguish. In a proper sense, anguish is defined as a feeling without an object, unlike anxiety that involves an object of anxiety. The reference matrix of alexithymia and intolerance to uncertainty would therefore lie in the absence of an object to be processed. This statement needs further clarification. The absence of the object towards which the operation is directed does not imply the non-existence of the same, as a failure to process and therefore the fact that the participant unable by alexithymia to process properly a coherent mental representation can only experience uncertainty. The absence of a specific object appears to be closer to anguish than to anxiety, whose distinction is clear in psychopathology and phenomenological studies concerning object-relations (114,139). Finally, in the light of these results, having acquired the presence of consistent mental health issues, alexithymia and intolerance to uncertainty in people with T1DM, a clear need for interventions emerges. Considering the data emerged and the confirmation of the hypotheses it was possible to realize the presence of intolerance to uncertainty, alexithymia and mental health difficulties. The necessary interventions should focus on the presence of these phenomena and their relationships. In particular, having emerged a consistent link between intolerance to uncertainty and alexithymia, it would be necessary to start psychotherapeutic programs useful for the treatment of phenomena themselves and their relationships. Given the close link between intolerance to uncertainty and alexithymia, interventions aimed at treating these variables are necessary. In line with the literature and therefore with the results of high-level studies, the predictive role of alexithymia must be taken into account. This requires intervention on the maladaptive variables, known for their role as well as predictive even inauspicious on biological treatments, compliance and the course of the main disease, as compared to the risk of occurrence of substantial mental health difficulties.

## Strengths and limitations

The current research paper entails some strengths and limitations. Starting with strengths, it represents research conducted through valid and well-known instruments investigating relevant dynamics. Studying psychological functioning of participants suffering from T1DM in a specific area, where a lack of knowledge of psychological issue is clear, represents a strength to be considered. Moreover, this first step would represent a scientific basis for the use of specific interventions based on solid psychodiagnostic evaluations. However, some limitations are present. First, the number of included participants is low; even if sufficiently represented, the population must be investigated widely and including other variables such as biochemical markers. Moreover, the absence of a control group represents a limitation for the study. Moreover, the reliance of self-report instruments introduces limitations to the study, due to a possible bias linked to inaccuracies in self-assessment. Subsequent research should take into account causal relations among the included variables, leading to the identification of predictors of negative outcomes. As a pilot study, the current manuscript represents a first step in psychological assessment of participants suffering from chronic diseases.

## Conclusions

Investigating mental health difficulties in adolescents affected by T1DM represents a necessary step to avoid the onset and the maintenance of associate pathological issues. In these terms, this paper included a sufficient number of adolescents suffering from a chronic condition capable of serious consequences. The emergence of the above-mentioned psychopathological phenomena constitutes the first necessary step for structuring specific interventions involving people with T1DM. Through the use of valid psychological assessment instruments, some concerning issues emerged within the sample. Beyond mental health difficulties, alexithymia and intolerance to uncertainty appeared to be consistently represented. Considering the maladaptive nature of these phenomena, the first step consisted of assessing adolescents suffering from T1DM in order to detect the extent of phenomena and to highlight the need for interventions. Current clinical practices should integrate classical medical procedures with psychological interventions in order to circumvent further pathological exacerbations. In these terms, psychological assessment and interventions based on evidence can be integrated into care for people with T1DM.

### Electronic supplementary material

Below is the link to the electronic supplementary material.

Supplementary Material 1

## Data Availability

The data that support the findings of this study are available from the corresponding author, EMM, upon reasonable request.

## Abbreviations

T1DM: Type 1 Diabetes Mellitus
