# Supplementary material for: Alexithymia, intolerance to uncertainty and mental health difficulties in adolescents with Type 1 diabetes mellitus
Source: Ital J Pediatr. 2024 May 17;50:99. doi: 10.1186/s13052-024-01647-4 (PMC11100042; doi:10.1186/s13052-024-01647-4)
Supplement: Supplementary file 1 — Supplementary Material 1 [file 13052_2024_1647_MOESM1_ESM.pdf]

SPRINGER NATURE

Change of authorship request form - Journals (pre-acceptance)

Important information. Please read.

- This form should be used by authors to request any change in authorship (adding/deleting authors) including changes in corresponding authors. This form should not be used for name changes. Please fully complete all sections. Use black ink and block capitals and provide each author's full name with the given name first followed by the family name.

- By signing this declaration, all authors guarantee that the order of the authors are in accordance with their scientific contribution, if applicable as different conventions apply per discipline, and that only authors have been added who made a meaningful contribution to the work.

- Please note, in author collaborations where there is formal agreement for representing the collaboration, it is sufficient for the representative or legal guarantor (usually the corresponding author) to complete and sign the Authorship Change Form on behalf of all authors, **next to the added/removed author(s)**. **(Complete Section 3, followed by Section 6.)**
  In author collaborations where there is no formal agreement for representing the collaboration and **there are more than 10 authors**, one may sign for all, provided the signer appends correspondence that attests that each of the authors have agreed to the change **and the added/removed authors sign the form**. **(Complete Section 3, followed by Section 6.)**

- Please note, we cannot investigate or mediate any authorship disputes. If you are unable to obtain agreement from all authors (including those who you wish to be removed) you must refer the matter to your institution(s) for investigation. Please inform us if you need to do this.

- If you are not able to return a fully completed form within **30 days** of the date that it was sent to the author requesting the change, we may have to withdraw your manuscript. We cannot publish manuscripts where authorship has not been agreed by all authors (including those who have been removed).

- Incomplete forms will be rejected.

- Please return/upload this form, fully completed, to the Journals Editorial Office. The Journal and/or Publisher will consider the information you have provided to decide whether to approve the proposed change in authorship. We may decide to contact your institution for more information or undertake a further investigation, if appropriate, before making a final decision.

SPRINGER NATURE

Change of authorship request form - Journals (pre-acceptance)

Section 1: Please provide the current title of manuscript

Manuscript ID no.: ITJP-D-23-00382R2

Title: Alexithymia, Intolerance to Uncertainty and Mental Health Difficulties in Adolescents with Type 1 Diabetes Mellitus

Section 2: Please provide the previous authorship, in the order shown on the manuscript before the changes were introduced. Please indicate the corresponding author by adding (CA) behind the name.

	First name(s)	Family name	ORCID or SCOPUS id, if available
1st author	Emanuele Maria	Merlo	https://orcid.org/0000-0001-6041-9186
2nd author	Rita	Tutino	
3rd author	Liam Alexander	MacKenzie Myles	https://orcid.org/0000-0002-7050-8112
4th author	Salvatore	Settineri	
5th author	Maria Carmela	Lia	
6th author	Domenico	Minasi	
7th author			
8th author			
9th author			
10th author			

Please use an additional sheet if there are more than 10 authors.

SPRINGER NATURE Springer Nature is one of the world's leading global research, educational and professional publishers, created in May 2015 through the combination of Nature Publishing Group, Palgrave Macmillan, Macmillan Education and Springer Science+Business Media.

SCANNER ODYSSEY APPS

SPRINGER NATURE

Change of authorship request form - Journals (pre-acceptance)

Section 3: Please provide a justification for change. Please use this section to explain your reasons for changing the authorship of your manuscript, e.g. what necessitated the change in authorship? Please refer to the (journal) policy pages for more information about authorship. Please explain why omitted authors were not originally included and/or why authors were removed on the submitted manuscript.

Dear Editorial Panel, the change refers to Salvatore Settineri. Since Prof. Settineri Retired, he's not interested anymore in publication and asked me to remove his name without any type of conflict. According to his request I provided for name removal and for the present change of authorship form. Finally, Salvatore Settineri Signed the present document in order to display his will to be removed. The whole process took place in absence of any conflict.

Section 4: Proposed new authorship. Please provide your new authorship list in the order you would like it to appear on the manuscript. Please indicate the corresponding author by adding (CA) behind the name. <u>If the Corresponding Author has changed, please indicate the reason under section 3</u>.

	First name(s)	Family name (this name will appear in full on the final publication and will be searchable in various abstract and indexing databases)	Affiliated institute	E-mail address
1st author	Emanuele Maria	Merlo (CA)	University of Messina	emerlo@unime.it
2nd author	Rita	Tutino	GOM, Pediatric Unit of Ospedali Riuniti	Tutinorita11@gmail.com
3rd author	Liam Alexanded	MacKenzie Myles	University of Oxford	liam.a.myles@outlook.com
4th author	Maria Carmela	Lia	GOM, Pediatric Unit of Ospedali Riuniti	mariacarmela.lia@ospedalerc.it
5th author	Domenico	Minasi	GOM, Pediatric Unit of Ospedali Riuniti	domenico.minasi@tiscali.it
6th author				
7th author				
8th author				
9th author				
10th author				

Please use an additional sheet if there are more than 10 authors.

SPRINGER NATURE Springer Nature is one of the world's leading global research, educational and professional publishers, created in May 2015 through the combination of Nature Publishing Group, Palgrave Macmillan, Macmillan Education and Springer Science+Business Media.

SCANNER ODYSSEY APPS

Change of authorship request form - Journals (pre-acceptance)

Section 5: Author contribution, Acknowledgement and Disclosures. Please use this section to provide a new disclosure statement and, if appropriate, acknowledge any contributors who have been removed as authors and ensure you state what contribution any new authors made (if applicable per the journal or book (series) policy). Please ensure these are updated in your manuscript - after approval of the change(s) - as our production department will not transfer the information in this form to your manuscript.

New acknowledgements: None

New Disclosures (financial and non-financial interests, funding): No funding.

New Author Contributions statement (if applicable per the journal policy): EMM, RT and DM made significant contribution to the conception and design of the study. EMM made a significant contribution to the design of the diagnostic protocol, statistical analysis of data and drafting of the manuscript. RT and EMM provided the acquisition of data useful for the statistical analysis. EMM and LAMM made a significant contribution to the interpretation of data and comparison with literature. LAMM, MCL and DM revised the manuscript and gave their approval. All authors contributed to the paper and approved its final version.

State 'Not applicable' if there are no new authors.

SPRINGER NATURE

Change of authorship request form - Journals (pre-acceptance)

Section 6: Declaration of agreement. *All* authors, unchanged, new and removed *must* sign this declaration.
(NB: Please print the form, (docu)-sign and return/upload a scanned copy. Please note that signatures that have been inserted as an image file are acceptable as long as it is handwritten. Typed names in the signature box are unacceptable.) *** Please delete as appropriate. Delete all of the bold if you were on the original authorship list and are remaining as an author.**

	First name	Family name		Signature	Date
1st author	Emanuele Maria	Merlo	I agree to the proposed new authorship shown in section 4 **/and the addition/removal*of my name to the authorship list** /and the proposed change in corresponding author	Merlo Emanuele Maria	15/02/2024
2nd author	Rita	Tutino	I agree to the proposed new authorship shown in section 4 **/and the addition/removal*of my name to the authorship list** /and the proposed change in corresponding author	Rita [illegible]	15/02/2024
3rd author	Liam Alexander	MacKenzie Myles	I agree to the proposed new authorship shown in section 4 **/and the addition/removal*of my name to the authorship list** /and the proposed change in corresponding author	[illegible]	15/02/2024
4th authors	Salvatore	Settineri	I agree to the proposed new authorship shown in section 4 **/and the addition/removal*of my name to the authorship list** /and the proposed change in corresponding author	[illegible]	15/02/2024
5th author	Maria Carmela	Lia	I agree to the proposed new authorship shown in section 4 **/and the addition/removal*of my name to the authorship list** /and the proposed change in corresponding author	[illegible]	15/02/2024
6th author	Domenico	Minasi	I agree to the proposed new authorship shown in section 4 **/and the addition/removal*of my name to the authorship list** /and the proposed change in corresponding author	[illegible]	15/02/2024
7th author			I agree to the proposed new authorship shown in section 4 **/and the addition/removal*of my name to the authorship list** /and the proposed change in corresponding author		

SCANNER ODYSSEY APPS

SPRINGER NATURE

Change of authorship request form - Journals (pre-acceptance)

	First name	Family name		Signature	Date
8th author			I agree to the proposed new authorship shown in section 4 **/and the addition/removal*of my name to the authorship list** /and the proposed change in corresponding author		
9th author			I agree to the proposed new authorship shown in section 4 **/and the addition/removal*of my name to the authorship list** /and the proposed change in corresponding author		
10th author			I agree to the proposed new authorship shown in section 4 **/and the addition/removal*of my name to the authorship list** /and the proposed change in corresponding author		

Please use an additional sheet if there are more than 10 authors.

In case of author collaborations with formal agreement:

	Name of consortium/consortia	First name	Family name		Signature	Date
Representative/legal guarantor				I agree to the proposed new authorship shown in section 4 **/and the addition/removal*of my name to the authorship list** /and the proposed change in corresponding author		

Both added/removed authors should complete the information in the first table under Section 6.

---- End of form ----

—
SPRINGER NATURE Springer Nature is one of the world's leading global research, educational and professional publishers, created in May 2015 through the combination of Nature Publishing Group, Palgrave Macmillan, Macmillan Education and Springer Science+Business Media.

SCANNER ODYSSEY APPS
